# Heterogeneity and clinical significance of *ETV1* translocations in human prostate cancer

**DOI:** 10.1038/sj.bjc.6604472

**Published:** 2008-07-01

**Authors:** G Attard, J Clark, L Ambroisine, I G Mills, G Fisher, P Flohr, A Reid, S Edwards, G Kovacs, D Berney, C Foster, C E Massie, A Fletcher, J S De Bono, P Scardino, J Cuzick, C S Cooper

**Affiliations:** 1 Institute of Cancer Research, Male Urological Cancer Research Centre, 15 Cotswold Road, Sutton, Surrey SM2 5NG, UK; 2 The Royal Marsden NHS Trust Foundation Hospital, Downs, Road, Sutton, Surrey SM2 5PT, UK; 3 Department of Mathematics and Statistics, Cancer Research UK Centre for Epidemiology, Wolfson Institute of Preventive Medicine, St Bartholomew's Medical School, Queen Mary, University of London, Charterhouse Square, London EC1M 6BQ, UK; 4 Department of Oncology, Uro-Oncology Research Group, Cancer Research UK Cambridge Research Institute, Li Ka Shing Centre, University of Cambridge, Robinson Way, Cambridge CB2 0RE, UK; 5 Molekulare Onkologie, Heidelberg Klinikum, Ruprecht-Karls-Universitat, Im Neuenheimer Feld 365, Heidelberg 69120, Germany; 6 The Orchid Tissue Laboratory, St Bartholomew's Hospital, London EC1A 7BE, UK; 7 Department of Pathology, The University of Liverpool, Duncan Building, Royal Liverpool University Hospital, Daulby Street, Liverpool L69 3GA, UK; 8 Department of Pathology, Memorial Sloan Kettering Cancer Center, New York, NY 10021, USA; 9 Department of Urology, Memorial Sloan-Kettering Cancer Center, 1275 York Avenue, New York, NY 10021, USA

**Keywords:** prostate cancer, *ETV1*, *ACSL3*, *ACSL3:ETV1* fusion

## Abstract

A fluorescence *in situ* hybridisation (FISH) assay has been used to screen for *ETV1* gene rearrangements in a cohort of 429 prostate cancers from patients who had been diagnosed by trans-urethral resection of the prostate. The presence of *ETV1* gene alterations (found in 23 cases, 5.4%) was correlated with higher Gleason Score (*P*=0.001), PSA level at diagnosis (*P*=<0.0001) and clinical stage (*P*=0.017) but was not linked to poorer survival. We found that the six previously characterised translocation partners of *ETV1* only accounted for 34% of *ETV1* re-arrangements (eight out of 23) in this series, with fusion to the androgen-repressed gene *C15orf21* representing the commonest event (four out of 23). In 5′-RACE experiments on RNA extracted from formalin-fixed tissue we identified the androgen-upregulated gene *ACSL3* as a new 5′-translocation partner of *ETV1*. These studies report a novel fusion partner for *ETV1* and highlight the considerable heterogeneity of *ETV1* gene rearrangements in human prostate cancer.

Recently, fusion of the prostate-specific androgen-regulated *TMPRSS2* gene to the ETS family transcription factor gene *ERG* was reported as a common event in prostate cancer (Tomlins *et al*, 2005, 2006; Clark *et al*, 2006; Iljin *et al*, 2006; Perner *et al*, 2006; Soller *et al*, 2006; Wang *et al*, 2006a; Yoshimoto *et al*, 2006; Hermans *et al*, 2006). Less frequently *TMPRSS2* becomes fused to *ETV1* and *ETV4*. In all these cases a *TMPRSS2-ETS* chimaeric gene is generated resulting in high-level expression of the fused 3′-*ETS* gene sequences. The reported incidence of *TMPRSS2:ETV1* fusion in these studies (1–2%) was, however, considerably lower than the observed incidence of *ETV1* gene overexpression (∼10% in prostate cancer). This prompted Tomlins *et al* (2007) to search for alternative mechanisms of *ETV1* overexpression. They identified five new 5′-fusion *ETV1* partners including the prostate-specific androgen-induced gene *SLC45A3*/*Prostein*, an endogenous retroviral element *HERV-K*, a prostate-specific androgen-repressed gene *C15orf21*, and a strongly expressed housekeeping gene *HNRPA2B1*. Additionally they found that in the two prostate cancer cell lines LNCaP and MDA-PCa2B, outlier expression of *ETV1* was caused through the entire *ETV1* gene becoming juxtaposed to sequences at 14q13.3–14q21.1. By characterising the expression of four contiguous genes within this region (*SLC25A21*, *MIPOL1*, *FOXA1* and *TTC6*), as well as that of *ETV1*, in LNCaP cells they demonstrated that this region exhibited prostate-specific expression that was coordinately regulated by androgens in a castration-resistant cell line model without formation of a fusion gene. In that study only single cases of each fusion were reported, with the exception of the juxtaposition of *ETV1* sequences to 14q13.3–14q21.1 where two cases were observed. It was therefore not possible to assess the relative importance of the different fusion partners in their small tumour set.

For *ERG* gene re-arrangements several studies have demonstrated links to clinicopathological indicators (Perner *et al*, 2006; Wang *et al*, 2006a; Demichelis *et al*, 2007; Nam *et al*, 2007). In a watchful waiting cohort of 111 patients, Demichelis *et al* (2007) reported a significant link between the presence of *ERG* alterations and prostate cancer-specific death. In a series of 165 patients who underwent prostatectomy, Nam *et al* (2007) found that the presence of a *TMPRSS2:ERG* fusion was associated with a greater probability of biochemical relapse. Additionally, we have recently demonstrated that loss of 5′-*ERG* sequences coupled with duplication of *TMPRSS2:ERG* fusion sequences predicts extremely poor cancer-specific survival independently of Gleason score and PSA level at diagnosis in a conservatively managed watchful waiting patient cohort (Attard *et al*, 2008). In contrast very little is known about the clinical significance of alteration at the *ETV1* gene locus.

To help identify biomarkers that may be of use in the management of men with prostate cancer, we have established a retrospective cohort of 429 men whose cancers were conservatively managed (Cuzick *et al*, 2006). Our analyses included centrally assigned Gleason scores determined by modern grading criteria, and allowed comparisons with several additional clinical parameters. In agreement with previous studies (Johansson *et al*, 2004; Albertsen *et al*, 2005; Cuzick *et al* 2006) we found Gleason score to be an important determinant of cancer-specific mortality, although baseline PSA and to a lesser extent stage of disease added further predictive value. The objective of the current study is initially to use our cohort of 429 conservatively managed prostate cancer cases to assess the potential clinical significance of *ETV1* gene alterations and in parallel to assess the relative frequency of each of the known *ETV1* fusion partners. As we found these partners to only account for ∼34% of all *ETV1* translocation events, we undertook 5′-RACE studies to identify novel *ETV1* fusion partners in our paraffin-embedded tumour samples.

## Results

### Fluorescence *in situ* hybridisation detection of *ETV1* fusions

**Figure 1:**
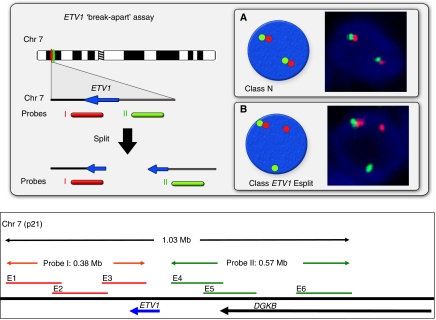
FISH detection of *ETV1* gene re-arrangements. Top: Interphase nuclei are hybridised to probes that detect sequences immediately 3′ to the *ETV1* gene (probe I, red) and immediately 5′ to the *ETV1* gene (probe II, green). The red and green signals are separated when an *ETV1* gene rearrangement occurs. (**A**) Signals from normal un-rearranged *ETV1* loci (class N). (**B**) Rearranged *ETV1* gene with separate red (3′) and green (5′) probes (class *ETV1* Esplit). Bottom: Map of the *ETV1* gene showing the position of the BACs used as probes in FISH assays. Probe I: E1 (RP11-27B1), E2 (RP11-138H16), E3 (CTD-2008I15) labelled with Cy3. Probe II E4 (RP11-905H4), E5 (RP11-621E24), E6 (RP11-115D14) labelled with FITC. The direction of transcription of genes at this locus are indicated by arrows.

We have used a fluorescence *in situ* hybridisation (FISH) *ETV1* gene ‘break-apart’ assay to screen for *ETV1* rearrangements on a Tissue Microarray (TMA) consisting of 945 trans-urethral resection of the prostate cancer cores from 429 patients. We used three overlapping BAC probes at the telomeric 3′-end (red) and three BAC probes at the centromeric 5′-end (green) of the *ETV1* gene (Figure 1). Normal *ETV1* loci are visualised in interphase nuclei as immediately adjacent green and red signals (Figure 1A, Class *ETV1* N). When rearrangements involving the *ETV1* gene were present, the 5′-centromeric and 3′-telomeric *ETV1* probes separated and were visible as lone red and green signals (Figure 1B, Class *ETV1* Esplit). These analyses identified *ETV1* gene rearrangements in cancer from 23 patients (5.4% of all cancers). An *ERG* gene break-apart assay, completed as previously described (Attard *et al*, 2008), demonstrated that an additional 155 cancers (36%) in this series contained *ERG* gene rearrangements, including one patient who had both *ERG* and *ETV1* rearrangements in distinct foci of cancer in the same prostate, as reported previously (Attard *et al*, 2008; Clark *et al*, 2008).

### Clinicopathological correlations

Tumour demographics and characteristics comparing patients with only *ETV1* gene rearrangements (22 cases) with patients who lacked both *ETV1* and *ERG* gene rearrangements (252 cases) are shown in Table 1. Correlations with clinical parameters demonstrated there were significant associations between the presence of *ETV1* gene re-arrangement and Gleason score (*P*=0.001), baseline PSA (*P*=<0.0001), clinical stage (*P*=0.017) and age (0.04). However, despite these links to indicators of more aggressive disease there was no evidence for a difference in overall and cancer-specific survival between those cancers harbouring *ETV1* gene alteration and those cancers retaining normal *ERG* and *ETV1* loci (Class N) (HR=1.48, CI=0.87–2.53, *P*=0.17 and HR=−1.48, CI=0.64–3.46, *P*=0.39 respectively) (Supplementary Figure 1).

### Heterogeneity of *ETV1* fusion partners

**Figure 2:**
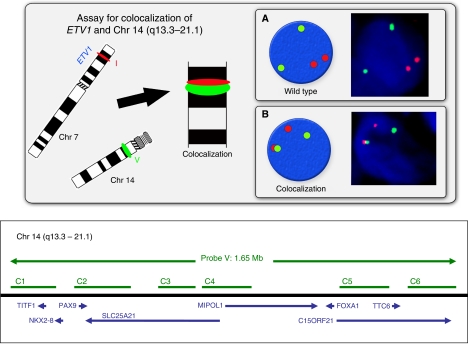
FISH detection of translocation of *ETV1* to chromosome 14(q13.3–21.1). Top: Interphase nuclei are hybridised to probes that detect sequences immediately 3′ to the *ETV1* gene on chromosome 7 (probe I, red) and a green probe (probe V) consisting of six BACS spanning the 14q13.3–21.1 region. (**A**). Red and green signals are normally separated. (**B**) Co-localisation of red and green probes indicate juxtaposition of chr 7 *ETV1* sequences with chr 14 (q 13.3–21.1). The lower panel shows the position of the BACs used for probe V: C1 (RP11-945C4), C2 (RP11-381L10), C3 (RP11-666J24), C4 (RP11-796F21), C5 (RP11-588D7), C6 (RP11-107E23) labelled with FITC. The relative position and direction of transcription of genes are indicated by the arrows.

We constructed a TMA block containing cores from all of the cancers harbouring *ETV1* re-arrangements (23 tumours) and six randomly selected cancers with an *ERG* gene rearrangement. We used slices of this TMA to carry out break-apart assays for the 5′-fusion partners previously identified by Tomlins *et al* (2005, 2006, 2007): namely *TMPRSS2*, *SLC45A3*, *HERV-K*, *C15orf21* and *HNRPA2B1* (Table 2). We also used FISH assays to confirm co-localisation of 3′-*ETV1* with 5′-sequences from each of the above partners as previously described by Tomlins *et al* (2007) (results not shown). To identify tumours with translocation of *ETV1* to the androgen-regulated prostate-specific region at 14q13.3–14q21.1 we co-hybridised a TMA slice with a 3′-*ETV1* FISH probe (red) and a FISH probe consisting of six BACs spanning the entire region of 14q13.3–q21.1 (green). Co-localisation of the red and green signals was taken as evidence of translocation of *ETV1* to this region (Figure 2). The FISH probes used in all of these assays are listed in Supplementary Table 1.

As expected, cancers with rearrangements of the *ERG* gene had fusions to 5′-*TMPRSS2* sequences. In contrast, none of the cancers with rearrangements of the *ETV1* gene exhibited fusions involving *TMPRSS2* or the *HERV-K* retroviral sequence. In four cancers 3′*-ETV1* exhibited fusion to 5′-*C15orf21* sequences, two contained translocation to 14q13.3–14q21.1, one contained fusion to *HNRPA2B1* and one contained fusion to *SLC45A5/Prostein* (Table 2). Thus only eight of the 23 cancers with re-arranged *ETV1* genes had known partners. The cancers containing fusion of 5′-*C15orf21* to 3′-*ETV1* sequences included the previously reported case containing *ERG* and *ETV1* rearrangements in distinct cancer foci of the same prostate (Clark *et al*, 2008). The recurrent fusions of the prostate-specific androgen-repressed gene *C15orf21* to 3′-*ETV1* sequences is of particular interest because Tomlins *et al* (2007) reported that this gene is not androgen driven, implying that tumours containing these fusion genes may exhibit resistance to androgen deprivation therapies. Joining of *ETV1* to individual partners was too uncommon to allow survival analysis for specific gene fusions. Of the four cases with a *C15orf21:ETV1* fusion, three are still alive and one died of unrelated causes.

### Fusion of the *ACSL3* gene to *ETV1* in human prostate cancer

**Figure 3:**
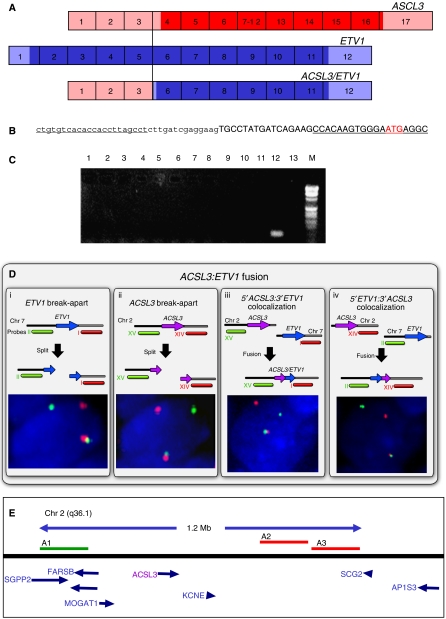
*ACSL3:ETV1* fusion. (**A**) *ACSL3* (red) and *ETV1* (blue) transcripts with ORFs in dark colour. Exons are numbered. A fusion transcript of *ACSL3* exon 3 fused to *ETV1* exon 6 was detected by 5′-RACE from exon 6 *ETV1* sequences in prostate cancer sample 23. The ORF shown was predicted using software at www.dnalc.org. (**B**) Sequence across the *ACSL3:ETV1* fusion boundary. Underlined regions indicate the position of primers used in RT–PCR to confirm the fusion. The predicted fusion gene initiation codon is indicated in red. *ACSL3* sequence is in lower case and *ETV1* sequence in upper case. (**C**) RT–PCR detection of an *ACSL3:ETV1* fusion transcript in RNA extracted from formalin-fixed paraffin-embedded prostate cancer samples: lanes 1–12 are *ETV1*-rearranged tumour samples, lane 12: tumour sample 23, lane 13 negative control. (**D**) FISH assays to confirm fusion of *ACSL3* with *ETV1*. Panel i: The *ETV1* break-apart assay utilises probes corresponding to 3′-*ETV1* sequences (red) and 5′-*ETV1* sequences (green) (see also Figure 1). A nucleus with separated red and green probes confirming rearrangement of *ETV1* is shown. Panel ii: The *ACSL3* break-apart assay hybridised the same TMA slice used in the *ETV1* break-apart assay to 3′-*ACSL3* sequences (red) and 5′-*ACSL3* sequences (green). These signals are coincident in the wild type, but are split on translocation of *ACSL3*. Comparison of the images in panels i and ii indicates co-localisation of 3′-*ETV1* with 5′-*ACSL3* and co-localisation of 5′-*ETV1* and 3′-*ACSL3*. This is confirmed by *ETV1-ACSL3* co-localisation assays (panel iii) demonstrating co-localisation of 3′-*ETV1* sequences (red) and 5′-*ACSL3* sequences (green) and (panel iv) demonstrating co-localisation of 3′-*ACSL3* sequences and 5′-*ETV1* sequences (red) in the same cell. Superimposition of the images in panels iii and iv confirms co-localisation of wild-type 3′-*ETV1* (panel iii) with 5′-*ETV1* (panel iv) and of wild-type 3′-*ACSL3* (panel iv) with 5′-*ACSL3* (panel iii). The genes and their direction of transcription are indicated by the arrowheads. (**E**) Map of the *ACSL3* gene showing the position of the BACs used as probes in FISH assays. Probe XV: A1 (RP11-157M20) labelled with FITC. Probe XIV: A2 (RP11-136M23) and A3 (RP11-749C15) labelled with Cy3. Probes XV and probes XIV correspond, respectively, to sequences immediately 5′ (green) and 3′ (red) to the *ACSL3* gene. Direction of gene transcription indicated by arrowheads.

We performed 5′-RACE to identify novel partners that are fused to 3′-*ETV1* sequences. Our studies were severely limited by the small amounts (50–200 ng) of poor quality RNA that could be prepared from the formalin-fixed tissue in this series. As obtainable RT–PCR products from these paraffin tissues were limited to ∼100–150 bp and the *ETV1* exon breakpoint in each sample was unknown, 5′-RACE–PCR had to be independently initiated from each of the known *ETV1* exon breakpoints in each sample, that is, exons 2, 4, 5 and 6. Using this strategy we successfully obtained a 5′-RACE fusion product from one RNA sample that contained an ex6 *ETV1* sequence fused to a 51 bp sequence of *ACSL3* ex3 sequence identifying *ACSL3* as a novel *ETV1* fusion partner. The structure of this *ACSL3* ex3:*ETV1* ex6 fusion is predicted to encode a truncated *ETV1* protein as shown in Figure 3A. The presence of the *ACSL3:ETV1* fusion was confirmed in this specimen by RT–PCR using 5′-*ACSL3* and 3′-*ETV1* primers (Figure 3B, C) and co-localisation by FISH of BAC probes corresponding to 5′-*ACSL3* sequences (green) and 3′-*ETV1* sequences (red) (Figure 3D, panel iii). An *ACSL3* break-apart FISH assay screen of the entire TMA containing the 23 cancers with rearrangement of the *ETV1* gene failed to identify additional cancers with this particular fusion. Like fusion to *TMPRSS2, HNRPA2B1, HERV-K or SLC45A5/Prostein* the fusion of 3′-*ETV1* sequences to 5′-*ACSL3* sequences is not a common event in this patient cohort. We have also demonstrated fusion of 5′-*ETV1* sequences with 3′-*ACSL3* sequences by FISH, indicating that the mechanism underlying formation of this fusion gene is a balanced translocation (Figure 3D, panel iv).

## Discussion

We have shown that the presence of *ETV1* gene locus rearrangements scored in a FISH-based assay is correlated with Gleason score, associated clinical stage and baseline PSA, but interestingly was not associated with poorer survival. Similar analyses of *ERG* gene alterations detected by FISH also demonstrate correlation to Gleason score, clinical stage and baseline PSA (Attard *et al*, 2008). However, in clinical outcome correlations only the presence of a duplication of rearranged *ERG* together with interstitial deletion of genomic sequences between the tandemly located *TMPRSS2* and *ERG* sequences was correlated with worse cancer-specific death (Attard *et al*, 2008). In analyses of alteration of the *ETV1* gene it was not possible to examine the relationship between survival and duplication of the *ETV1* foci because duplications were only found in five of the 23 cases examined (one *C15orf21* fusion, one chr14 co-localisation, and three with unknown partners).

Previous studies have each reported single cancers with an *ETV1* rearrangement (Tomlins *et al*, 2005, 2006; Hermans *et al*, 2006) with the exception of Tomlins *et al* (2007) who reported four clinical cases. Our study therefore represents the largest single series of primary prostate cases with an *ETV1* rearrangement. Our study confirms previous observations that *ETV1* may form a fusion gene with a variety of partners and shows that each individual fusion is relatively rare. Importantly, we show that the known fusion partners, including the novel *ACSL3:ETV1* fusion gene, only account for 39% of cancers with an *ETV1* rearrangement and it is therefore likely that many new partner genes remain to be identified.

The protein encoded by the *ACSL3* gene is an isozyme of the long-chain fatty-acid coenzyme A ligase family that converts free long-chain fatty acids into fatty acyl-CoA esters, and thereby plays a key role in lipid biosynthesis and fatty acid degradation. Insights into the regulation of *ACSL3* expression arise from expression array data in which the LNCaP cell line was treated with the synthetic androgen R1881. In two independent expression array data sets, *ACSL3* was upregulated by androgen treatment (Hendriksen *et al*, 2006; Wang *et al*, 2006b). One study showed *ACSL3* upregulation at time intervals of 2, 4, 6 and 8 h following androgen treatment (Hendriksen *et al*, 2006) and another study showed *ACSL3* upregulation after 16 h (Wang *et al*, 2006b). Expression of *ACSL3* was also elevated in a panel of ‘androgen-sensitive’ (LAPC-4, LNCaP, MDA PCa2a, MDA PCa2b, and 22Rv1) versus ‘androgen-insensitive’ (PPC1, PC3, and DU145) prostate cancer cell lines (Zhao *et al*, 2005; Tomlins *et al*, 2007). Expression of *TMPRSS2* and *SLC45A3* follow the same pattern within these datasets (Zhao *et al*, 2005). *ACSL3* transcription can also be activated by oncostatin via the ERK-signalling pathway (Zhou *et al*, 2007) suggesting alternative means of regulation.

These observations raised the question of whether there are any androgen receptor (AR) binding sites capable of explaining the expression levels of *ACSL3* and of the other known *ETV1* partner genes. A number of groups have recently published AR ChIP-chip studies mapping AR-binding sites within the human genome (Bolton *et al*, 2007; Massie *et al*, 2007; Takayama *et al*, 2007; Wang *et al*, 2007). Wang *et al* (2007) identified a functional AR-binding site 13.5 kb upstream of the *TMPRSS2* gene (Wang *et al*, 2007). The closest AR-binding sites for the other genes involved in *ETS* gene fusions varied from 60 kb to 1.5 Mb, although in the absence of genome-wide AR ChIP data it is possible that other AR-binding sites occur outside of the current coverage (Supplementary Table 2). Both Massie *et al* (2007) and Wang *et al* (2007) have proposed mechanisms for AR recruitment to subsets of target sequences through associations between the AR and other transcription factors for example, GATA-2, OCT11, FOXA1 and ETS1.

In conclusion our studies report a novel fusion partner for *ETV1* and highlight the wide heterogeneity in the range of the *ETV1* fusion partners. Interestingly fusion to the androgen repressed gene *C15orf21* was the most common event suggesting the existence of a significant subgroup of cancers that may not respond in a conventional manner to androgen withdrawal therapies.

## Materials and methods

### Patient cohort and tissue microarrays

TMAs were constructed from 429 unselected transurethral resection of the prostate specimens taken from patients managed with no initial treatment or hormone treatment in a cohort of conservatively managed men with prostate cancer (Cuzick *et al*, 2006). The median age of diagnosis was 70 years (49–76 years) and the median follow up was 91 months (3–173 months). Most men were diagnosed after the age of 65 years. National approval for the collection of the cohort was obtained from the Northern Multi-Research Ethics Committee followed by local ethics committee approval at each of the collaborating hospital trusts. This work was approved by the Clinical Research and Ethics Committee at the Royal Marsden Hospital and Institute of Cancer Research.

### Tissue microarrays

TMAs were constructed in 35 × 22 × 7 mm blocks of Lamb paraffin wax using a manual tissue microarrayer (Beecher Instruments, Sun Prairie, WI, USA). Up to four tumour cores of 600 *μ*m diameter were taken from each prostate. Reassignment of areas of ‘cancer’ or ‘normal’ in each core was carried out on the basis of histopathological examination of haematoxylin and eosin and p63 and AMACR-stained sections that flanked the TMA slice used for FISH studies. The morphological criteria for selection of ‘normal’ and ‘malignant’ prostatic epithelium conformed to previously published definitions (Foster, 2000; Foster *et al*, 2000, 2004). ‘Hyperplasia’, ‘dysplasia’ and ‘PIN’ were not scored in this study.

### FISH studies

TMA sections (4 *μ*m) were cut onto SuperFrostPlus glass slides (VWR International, Poole, UK). Fluorescence *in situ* hybridisation studies, labelling of BACs including preparation of slides, probes and washing were all carried out as described previously (Attard *et al*, 2008; Clark J *et al*, 2008)

### 5′ RACE RT–PCR from paraffin-embedded tissue

RNA was extracted from a 600 *μ*m core of paraffin-embedded tumour tissue using the RecoverAll kit as manufacturer's instructions (Ambion, UK, cat. AM1975). A total of 25–100 ng of RNA was reverse transcribed using 50 U Superscript III (Invitrogen, Paisley, UK) and 10 ng random nonamers in a 25 *μ*l reaction as manufacturer's instructions. cDNAs were treated with 0·3 *μ*l (1 U) RNAase-H (20 min), then extracted with one volume of phenol/chloroform (1 : 1 v v^−1^), then one volume of chloroform and then precipitated with 500 ng glycogen (Sigma UK, G1767), rinsed in 80% v v^−1^ ethanol, and resuspended in 15 *μ*l water. Second strand cDNA synthesis was then carried out: Klenow buffer plus 0.3 *μ*l of 10 *μ*M TAGrandom primer (GACTCGAGTCGACATCGAIIINNNNNN where I is Inosine) were added, heated to 70°C 5 min, cooled to room temperature and 40 U Klenow added (25°C 10 min, 30°C 10 min 37°C 1 h, 75°C 10 min). 1 *μ*l (2–8 ng) was used to seed a 25 *μ*l PCR mix with 0.25 U Platinum Taq (Invitrogen) plus an *ETV1* exon 6 primer GCCTCATTCCCACTTGTGG, 50 rounds, 61.5°C annealing temperature. TAG primer (GACTCGAGTCGACATCGA) was then added and the reaction continued for 40 rounds at 59°C annealing. 0.25 *μ*l of this was used to seed a nested PCR using TAG primer and *ETV1* exon 6 primary nest primer TTCCCACTTGTGGCTTCTG, 59°C annealing, 40 rounds. 0.25 *μ*l of this was then used to seed two PCRs, the first containing TAG primer and secondary nest ETV1 primer cccacttgtggcttctgatc, and the second containing TAG primer alone, 40 rounds 59°C. RT–PCR products were run on 2% agarose TAE gels. RACE products were subcloned using the TA cloning kit (Invitrogen) and sequenced. Sequences were searched at the human genome map web site (http://genome.ucsc.edu).

## Figures and Tables

**Table 1:** *ETV1* classification and revised Gleason score^a^

**Gleason score**	**Class N**	**Class *ETV1* Esplit**	**Total**
4	2	0	2
5	11	1	12
6	130	2	132
7	59	11	70
8	27	3	30
9	20	5	25
10	2	0	2
Unknown	1	0	1
Total	252	22	274

a Revised Gleason score for cancers lacking *ERG* and *ETV1* rearrangements (class N) is compared to cancers with rearrangement of *ETV1* (class *ETV1* Esplit).

**Table 2:** Frequency of detection of known *ETV1* translocations^a^

**Class of *ETV1* rearrangement**	**Number of cases (% of total number of *ETV1*-rearranged cancers)**
Fusion with *C15ORF21*	4 (18%)
Rearrangement to 14q13.3–14q21.1	2 (9%)
Fusion with *ACSL3*	1 (4.5%)
Fusion with *HNRPA2B1*	1 (4.5%)
Fusion with *SLC45A5/Prostein*	1 (4.5%)
Fusion with *HERV-K*	0
Fusion with *TMPRSS2*	0

a The number of cases of *ETV1* rearrangement involving each of the six previously described translocation partners namely *C15ORF21*, 4q13.3–14q21.1, *HNRPA2B1,* *SLC45A5/Prostein,* *HERV-K* and *TMPRSS2*, plus our discovery of translocation with *ACSL3* in our series of 23 *ETV1*-rearranged cancers.
