# Supplementary figures and images for: Heterogeneity and clinical significance of ETV1 translocations in human prostate cancer
Source: Br J Cancer. 2008 Jul 1;99(2):314–20. doi: 10.1038/sj.bjc.6604472 (PMC2480965; doi:10.1038/sj.bjc.6604472)

## Slide 1
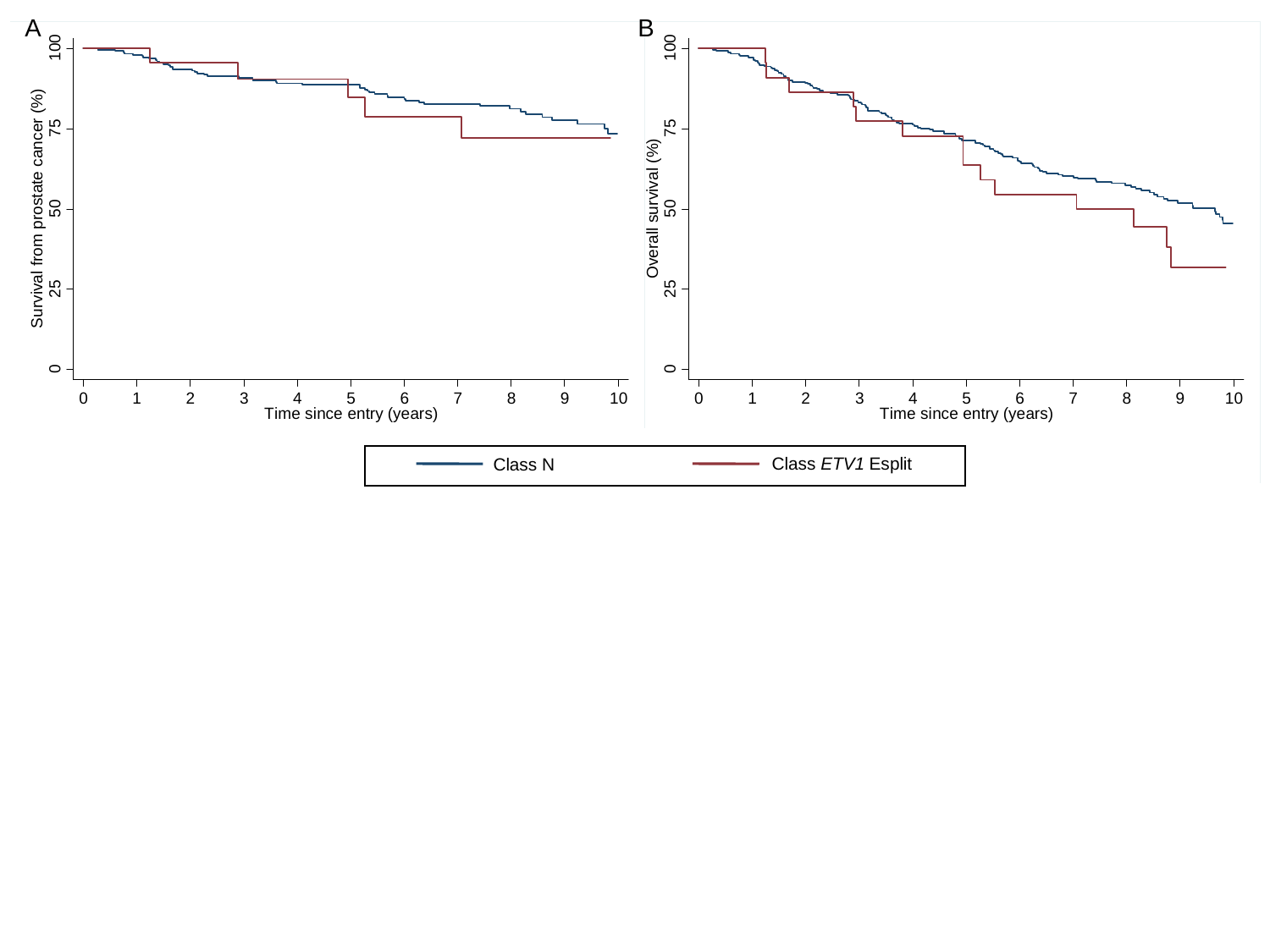

A
B
Class ETV1 Esplit
Class N

Supplement: Supplementary Figure 1 [file 6604472x1.ppt]
