# Supplementary material for: Heterogeneity and clinical significance of ETV1 translocations in human prostate cancer
Source: Br J Cancer. 2008 Jul 1;99(2):314–20. doi: 10.1038/sj.bjc.6604472 (PMC2480965; doi:10.1038/sj.bjc.6604472)
Supplement: Supplementary Legend [file 6604472x2.doc]

Supplementary Figure 1. Kaplan–Meier analyses comparing prostate cancer patient outcomes. The graphs compare cancers harbouring *ETV1* gene alteration (class *ETV1* Esplit) with cancers lacking both *ETV1* and *ERG* gene alterations (class N). (A) Cause-specific survival. (B) Overall survival.
