# Supplementary material for: Heterogeneity and clinical significance of ETV1 translocations in human prostate cancer
Source: Br J Cancer. 2008 Jul 1;99(2):314–20. doi: 10.1038/sj.bjc.6604472 (PMC2480965; doi:10.1038/sj.bjc.6604472)
Supplement: Supplementary Table 1 [file 6604472x3.doc]

**Supplementary Table 1**. **BACs comprising each Fluorescence in situ hybridisation probe.**

| **Probe #** | **Gene/region** | **Localisation** | **Probe (Accession number)** |
| --- | --- | --- | --- |
| I | ETV1 | 3’ | RP11-27B1, RP11-138H16, CTD-2008I15 |
| II | ETV1 | 5’ | RP11-905H4, RP11-621E24, RP11-115D14 |
| III | C15ORF21 | 3’ | RP11-626F7 |
| IV | C15ORF21 | 5’ | RP11-1125I3 |
| V | Chr14(q 13.3 – 21.1) | Chr 14  (q13.3 – 21.1) | RP11-945C4, RP11-381L10, RP11-666J24, RP11-796F21, RP11-588D7, RP11-107E23 |
| VI | SLC45A5/Prostein | 3’ | RP11-379J10, RP11-1143H2 |
| VII | SLC45A5/Prostein | 5’ | RP11-219P13 |
| VIII | HNRPA2B1 | 3’ | RP11-91F20 |
| IX | HNRPA2B1 | 5’ | RP11-1006O2, RP11-379M24 |
| X | HERV-K _22(q11.23) | 3’ | RP11-71G19 |
| XI | HERV-K _22(q11.23) | 5’ | RP11-947A12 |
| XII | TMPRSS2 | 3’ | RP11-114H1, 11:RP11-662D5 |
| XIII | TMPRSS2 | 5’ | G248P89444D12, G248P800876A1, G248P8239C5, RP11-35C4, RP11-282I20 |
| XIV | ACSL3 | 3’ | RP11-136M23, RP11-749C15 |
| XV | ACSL3 | 5’ | RP11-157M20 |
