# Supplementary material for: Heterogeneity and clinical significance of ETV1 translocations in human prostate cancer
Source: Br J Cancer. 2008 Jul 1;99(2):314–20. doi: 10.1038/sj.bjc.6604472 (PMC2480965; doi:10.1038/sj.bjc.6604472)
Supplement: Supplementary Table 2 [file 6604472x4.doc]

**Supplementary Table 2. ^†^Androgen receptor (AR) binding sites (AREs) in the genomic regions of ETS fusion partner genes.**

| **Target** | **Distance to nearest ARE based on ChIP-chip datasets** | **Predicted AREs within 5kb of start site (repeat-masked JASPAR searches)** |
| --- | --- | --- |
| *TMPRSS2* | 13.5kb upstream | None |
| *Herv-K* | 70kb and 100kb upstream | None |
| *ACSL3* | 350kb upstream within the promoter of *SGPP2* | 4kb upstream |
| *SLC45A3* | 60kb upstream in the promoter of *NUCKS1* | 1.5kb upstream |
| *HNRPA2B1* | 800kb upstream in the promoter of *SCAP2* | None |
| *MIPOL1* region | 1.5Mb upstream | 1kb upstream |
| *C15orf21* | 100kb and 200kb upstream in the promoters of *SPATA5L1* and *SHF* | None |

**^†^**Mapped AR binding sites were identified from four published AR chromatin immunoprecipitation (ChIP)-on-chip studies . AR ChIP-chip data were retrieved, converted to GFF format and uploaded to the UCSC Genome Browser. Genomic loci around the ETS fusion partner genes were examined for the nearest AR binding sites found in any of these studies. In a parallel approach we searched for predicted AR binding sites in the 5kb promoter sequences of the ETS fusion partner genes. Sequences corresponding to the 5kb regions immediately upstream of the transcriptional start site for each gene were retrieved from the UCSC Genome Browser and these sequences were analysed for the occurrence of the consensus ARE sequence using JASPAR.
